# Advocating for Our Children: An Initiative Utilizing Verbal and Video Education to Increase Adverse Childhood Experiences Questionnaire Form Response Rate

**DOI:** 10.1097/pq9.0000000000000801

**Published:** 2025-03-06

**Authors:** Madison R. Tyle, Shainal Gandhi, Nikhita Nookala, Kelly A. Campbell, Melissa Chow, Marilyn Torres, Sarah A. Commaroto, Monica Khadka, Emily Coughlin, Vinita Kiluk

**Affiliations:** From the * Morsani College of Medicine, University of South Florida, Tampa, Florida; † Department of Pediatrics, University of South Florida, Tampa, Florida.

## Abstract

**Introduction:**

Negative experiences in childhood, Adverse Childhood Experiences, significantly increase the risk of adverse health outcomes in adulthood. Obtaining a better understanding of the experiences a child has been through during development allows providers to connect them with resources to improve health outcomes.

**Methods:**

We performed problem identification via PubMed and the Florida Department of Health web page. We used the plan-do-study-act (PDSA) quality improvement method. Intervention one involved teaching clinic staff about distributing the Adverse Childhood Experiences Questionnaire (ACE-Q) form during well-check visits. Intervention two involved a video education tool to explain the purpose and importance of the ACE-Q to caretakers. We conducted a retrospective chart review at the 17 Davis and HealthPark clinics 3 months preceding each PDSA cycle. We analyzed the data to assess the response rate to the ACE-Q before and after each cycle.

**Results:**

The educational initiatives increased the response rate to the ACE-Q form in both PDSA cycles. The ACE-Q was significantly more likely to be filled out after the first (19.2% in pre versus 24.8% in post, *P* < 0.001) and second PDSA cycles (15% in pre versus 45.2% in post, *P* < 0.001).

**Conclusions:**

Verbal and video education models can increase the response rate to the ACE-Q. Response collection is valuable for identifying and supporting patients at the highest risk for poor health outcomes. Future studies would benefit from addressing low view counts on video interventions, standardizing ACE-Q score assessment, and implementing sustainable measures.

## INTRODUCTION

It is proven that exposure to adverse events in childhood affects development negatively.^1–3^ In 1998, Kaiser Permanente’s adverse childhood experiences (ACEs) study described for the first time the relationship between disease in adulthood and childhood exposures to abuse and household dysfunction.^4^ This study looked at several ACEs, including psychological, physical, and sexual abuse, as well as exposure to substance abuse, mental illness, violent treatment of the mother, or criminal behavior in the household. The results revealed a strong relationship between these childhood experiences and several of the leading causes of death in adulthood.^4^

Since the original 1998 study, a wide array of research revealed the link between high ACEs and poor adult health outcomes. ACEs strongly correlate to an increased risk for depression, obesity, diabetes, use of tobacco products, alcohol use, cancer, heart disease, respiratory illness, sexually transmitted infections, delinquency, and self-directed violence.^5–8^ However, research has shown that early intervention can improve health outcomes.^9,10^

Screening for ACEs is feasible and preferred during well-child checks.^11–13^ To assess ACEs in the pediatric clinic setting, providers can utilize the ACE Questionnaire (ACE-Q) to screen for history of abuse, neglect, and household dysfunction in their patients. However, to understand the breadth of adversity a child has experienced, their parent or legal guardian must fill out this form on their behalf. Filling out the ACE-Q can be challenging, as some parents view the questions and topics addressed in the form as invasive and triggering. Some barriers to completing the ACE-Q reported in the literature include emotional unreadiness to disclose, lack of trust in the provider or trust not yet established, fear of child protective services involvement, immigration implications, privacy concerns, and feelings of embarrassment, guilt, and/or shame.^14^

ACE-Q form completion is of particular interest to our local community in Hillsborough County. According to the Florida Department of Health, in 2023, the rate per 100,000 of Children Experiencing Child Abuse (ages 5–11 y) in Hillsborough County was 585.9 compared with Florida at 431.5. Hillsborough County is in the third quartile for this measure. Thus, Hillsborough County has more cases of children experiencing child abuse (ages 5–11 y) than one-quarter of the counties in Florida.^15^ One way to assess for child abuse, child neglect, and household dysfunction in the pediatric clinical setting is the ACE-Q form. Thus, it is crucial for families and caregivers to adequately understand the purpose of this questionnaire so that they can accurately fill it out on behalf of their children and allow healthcare providers the opportunity to intervene early when necessary.

This QI project aimed to use two education initiatives to increase ACE-Q distribution and response rates. We introduced each education initiative during a separate PDSA cycle. In the first intervention, healthcare personnel received training on the ACE-Q, which included implementing a system to increase distribution. In the second initiative, we used video education to educate caregivers of pediatric patients on the importance of screening for ACEs in the pediatric clinic setting. We conducted both initiatives to increase the response rate to the ACE-Q form at the provider continuity pediatric clinics in Tampa, FL.

## METHODS

### Context

Tampa General Hospital and USF Health are affiliated with two outpatient general pediatrics clinics, HealthPark and 17 Davis. These locations were the primary sites for the interventions described below. Clinic staff includes various attending physicians, residents, and nurse practitioners, and the quality improvement team responsible for the interventions consists of several residents, medical students, and an attending.

**Fig. 1.**
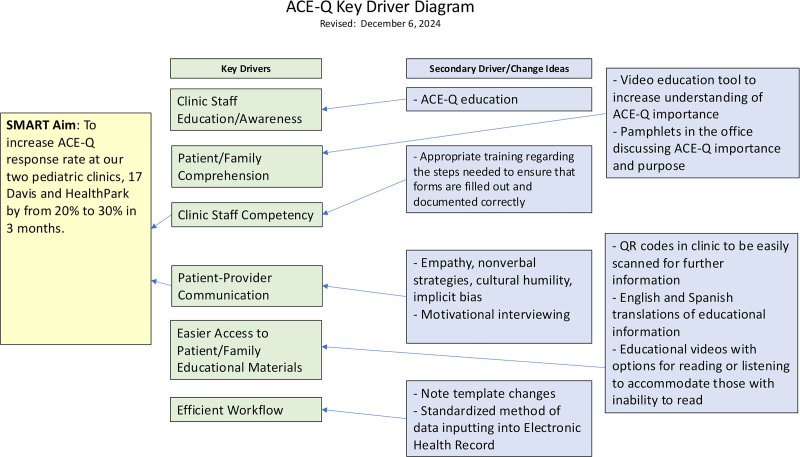
Key drivers for PDSA cycles 1 and 2.

We conducted a brief review of the literature and local statistics using the PubMed search engine and the Florida Department of Health web page to understand the severity of the problem in our local community. From there, we discussed barriers to form completion and documentation to create the resulting list of key drivers that document the factors that impair ACE-Q completion. We repeated this process following the completion of the first intervention. We separated the key drivers and used them to create and guide two interventions for PDSA cycles 1 and 2 (Fig. 1).

Reviewing charts included utilizing the electronic medical record (EMR), Epic, at both clinic sites (Epic Systems, Inc., Verona, Wis.). All well-child checks were reviewed at each of the clinics. The number of charts reviewed each month was recorded.

### Interventions

**Fig. 2.**
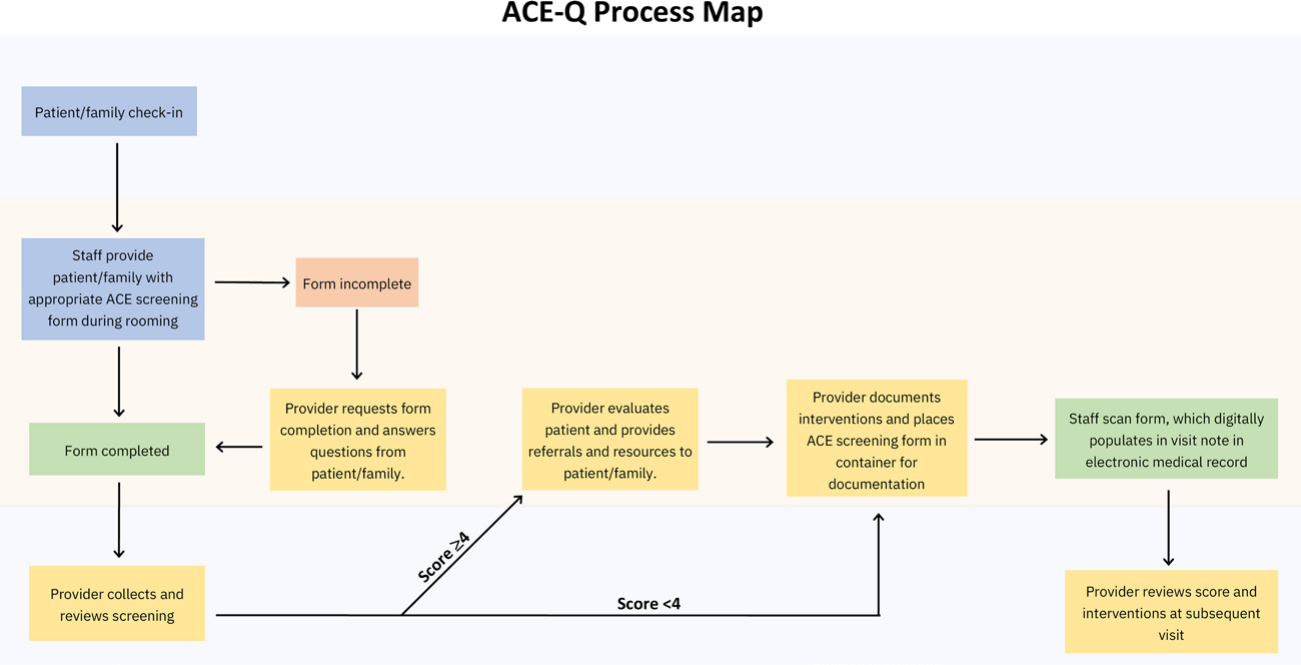
Process map.

The research team utilized two interventions in this QI project to educate healthcare personnel and nonhealthcare professionals/caregivers. We introduced each education initiative during a separate PDSA cycle. On January 17, 2022, the first PDSA cycle began with educating the front-desk staff, nursing staff, attendings and resident physicians at 17 Davis and HealthPark continuity clinics in the Tampa Bay area on the importance and need to distribute the ACE-Q form to patients’ caregivers during the well-check visits. The intervention was primarily verbal, explaining to staff the reasons behind collecting the ACE-Q forms and a clear outline of the steps needed to ensure that forms are filled out and documented correctly. The goal of this initiative was principally to raise awareness among clinic healthcare personnel regarding ACEs and their effects on patient health outcomes. Although we primarily directed this training at healthcare providers, it also included implementing a system to distribute and collect ACE-Q forms, which could be documented in the EMR. The system involved a streamlined process for passing out the ACE-Q after patient rooming. Based on the provider’s initial review of form completion, further steps could be taken to encourage ACE-Q form completion. We outlined this system in the process map in Figure 2.

**Fig. 3.**
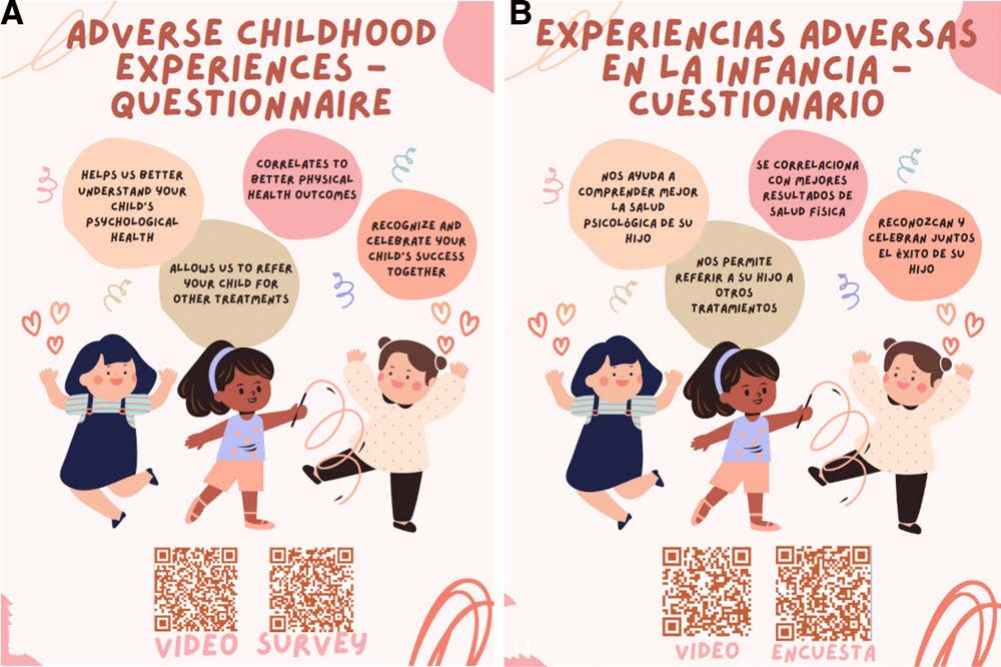
A and B, English and Spanish posters hung in the clinics during PDSA cycle no. 2.

The following year, we implemented a second intervention during the next PDSA cycle, which sought to raise awareness among the caretakers of children in the clinic. The first author created a video education tool to explain the purpose and importance of the ACE-Q using simplistic verbiage for caretaker education. (See Video [online], which describes ACE-Q animated video.) (The ACE-Q Form © 2022 by Madison Tyle is licensed under CC BY-NC-ND 4.0.) YouTube video counts were used to track the number of views on the video. We included a QR code containing a link to the video education tool on posters hung in well-exam visit rooms and waiting areas at 17 Davis and Health Park clinics on February 15, 2023. The posters also included brief information about the benefits of the ACE-Q. The poster’s English and Spanish versions were used (Fig. 3A, B). The goal of the video was to educate the caretakers of pediatric patients on the value of filling out the ACE-Q forms.

**Video 1:** 

### Measures

The primary outcome measure was the percentage of ACE-Q forms completed and documented. We obtained this measurement by comparing the number of completed and documented screens to the total number of well-child visits each month. All well-child visits were reviewed each month. We conducted a subgroup analysis to assess each clinic’s percentages.

### Data Collection

We collected data retrospectively via chart review through the Epic EMR for patients who visited the 17 Davis and HealthPark clinic sites for a well-child visit between the various intervention dates. We documented the data using Research Electronic Data Capture (REDCap) software to build a clinical database. Collected data included patient demographics (age, sex, and race), date of visit, and the response to the ACE-Q. The response data included whether the patient/guardian responded to the questionnaire, whether they filled out the questionnaire during previous well visits, the numerical score result, whether the parent/guardian noted any symptoms, and whether the healthcare team made any referrals to various community resources.

We collected the first data set between October 7, 2021, and April 9, 2022, representing data 3 months before and 3 months after the intervention on January 17, 2022. Data collected from October 7, 2021, to January 17, 2022, are categorized as “preintervention, round 1.” Data collected from January 17, 2022, to April 9, 2022, are categorized as “postintervention, round 1.” We repeated this process the following year between October 15, 2022, and May 17, 2023, representing data 3 months before and 3 months after the intervention on February 15, 2023. Data collected from October 15, 2022, to February 15, 2023, are categorized as “preintervention, round 2.” Data collected from February 15, 2023, to May 17, 2023, are categorized as “postintervention, round 2.” No data were collected between April 9, 2022, and October 15, 2022, as this was the period between the 2 PDSA cycles.

### Statistical Methods

We analyzed data using descriptive statistics and compared response rates using Pearson’s chi-square tests. We completed the analysis using SPSS version 29. Run charts were generated using Excel, with the number of charts reviewed monthly on the x-axis.

## RESULTS

During the first round of the intervention, 2,254 charts were reviewed, with 584 from the HealthPark clinic and 1,670 from the 17 Davis clinic. During the second round of the intervention, 1,608 charts were reviewed, with 874 from the HealthPark clinic and 734 from the 17 Davis clinic. Demographic information can be found in Table 1. The summary of results is in Table 2.

**Table 1.** Summary of Demographic Data (Sex, Race, and Age) for Collected Data

Sex (n = 5,517)	
Male	2,890 (52.4%)
Female	2,621 (47.6%)
Race (n = 4,413)	
White	1,921 (34.8%)
Black	2,047 (37.1%)
Asian	162 (2.9%)
American Indian	11 (0.2%)
Native Hawaiian	5 (0.1%)
More than one race	267 (4.8%)
Age, y (n = 5,508)	
<1	1,646 (29.8%)
≥1	3,862 (70.0%)

**Table 2.** Comparison of % Caretakers Who Filled Out the ACE-Q Form before and after Each of the Interventions at the 17 Davis and HealthPark Clinics

	No. Caretakers Who Filled Out the ACE-Q Form			
	Pre-PDSA Cycle 1	Post-PDSA Cycle 1	Pre-PDSA Cycle 2	Post-PDSA Cycle 2
17 Davis	129/917 (14.1%)	292/1,267 (23.0%)	47/606 (7.8%)	96/457 (21.0%)
HealthPark	135/459 (29.4%)	145/493 (29.4%)	142/621 (22.9%)	425/696 (61.1%)
Combined Data	264/1,376 (19.2%)	437/1,760 (24.8%)	189/1,227 (15.4%)	521/1,153 (45.2%)

The ACE-Q was significantly more likely to be filled out after the intervention in year 1 (*P* < 0.001). Before the intervention, HealthPark was significantly more likely to have ACE-Q filled out than 17 Davis (*P* < 0.001). After the intervention, Health Park was also significantly more likely to have ACE-Q filled out than 17 Davis (*P* = 0.006). In year 1, HealthPark did not show significant improvement postintervention (*P* = 1.000), whereas 17 Davis did show improvement (*P* < 0.001).

During the second PDSA cycle, the video intervention had 38 YouTube view counts. In year 2, the ACE-Q was also significantly more likely to be filled out after the intervention (*P* < 0.001). Before the intervention, HealthPark was significantly more likely to have ACE-Q filled out than 17 Davis (*P* < 0.001). After the intervention, HealthPark was also significantly more likely to have ACE-Q filled out than 17 Davis (*P* < 0.001). In year 2, HealthPark and 17 Davis significantly improved after the intervention.

**Fig. 4.**
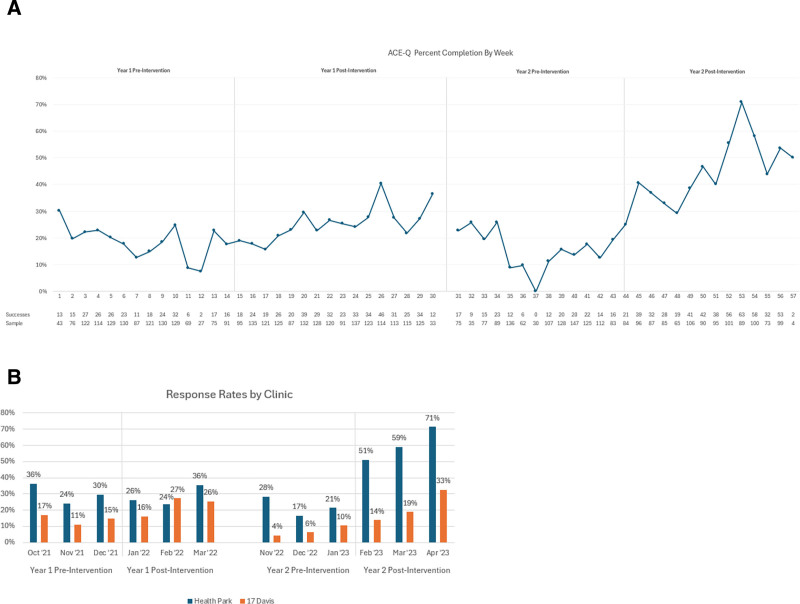
Posters hung up in pediatric clinics in English and Spanish during PDSA cycle no. 2 to allow access to the video material via QR code. A and B, Line graph for ACE-Q response rate and subgroup analysis.

A line plot of the data results for PDSA cycles 1 and 2 is shown in Figure 4A. Figure 4B presents a subgroup analysis chart.

Throughout the chart review, we standardized the method by incorporating the ACE-Q scoring template in the well-child visit note template used in EPIC. Still, the interpretation and use of the ACE-Q score in the assessment and plan varied from provider to provider.

## DISCUSSION

### Impact of Interventions on ACE-Q Response Percentage

The total response rates for the ACE-Q significantly improved from the preintervention and postintervention phases of the 2022 and 2023 intervention periods. We further analyzed these improvements at the data collection site. In year one, after the healthcare personnel education intervention (intervention 1), the ACE-Q response rate at HealthPark did not change significantly. In contrast, the ACE-Q response rate at the 17 Davis clinics did show a significant improvement. However, the ACE-Q response rate at HealthPark was still significantly higher than at the 17 Davis clinic throughout both periods. In year 2, both clinics significantly improved their ACE-Q response rate after the video intervention. HealthPark had a significantly higher response rate to the ACE-Q than 17 Davis in both periods. This result could be attributed to differences in several variables, such as characteristics of the front-desk staff at both clinics, differences in education on documentation between residents, advanced practice providers, and/or rotating students, or visibility of posters for the video intervention in both clinics; these are factors that can be difficult to standardize.

### Importance of Consistent ACE-Q Screening

ACE-Q screening yearly at routine visits can be a useful tool to document changing factors and trauma incidences in a child’s life and provide timely referrals to needed psychological services and positive parenting resources, such as HOT DOCs (University of South Florida, Tampa, FL) or Triple P parenting programs (Triple P International, Brisbane, Australia) in the Tampa Bay area.^10,16,17^ Annual well-child visits are a great opportunity to conduct these screenings and collect baseline data and changes due to their regularity and the absence of other acute issues to address immediately. Children who are in low-income households and who have fewer resources, such as access to healthy food options, transportation, education, and so on, also have disproportionately high rates of emotional and behavioral health problems, as well as disproportionately high ACE scores.^18,19^ This is especially important to address, as childhood ACE scores can predict the incidence of depression, PTSD, and methods of coping with adversity in adulthood.^5^ Collecting baseline data for our patients starting at one year of age can serve the dual purpose of letting physicians and advanced practice providers know which patients are at the highest risk of physical or mental symptoms of ACEs and trauma exposure. These data can be used to plan for additional staff, such as social workers, case managers, and care coordinators, who can assist with prioritizing patients and providing additional resources. Tracking ACE-Q scores as children get older can be a useful tool for clinicians to evaluate changes in a child’s life that can impact their current and adult health, and it provides a standard and validated tool to identify the events in a child’s life that are likely to impact them. Therefore, developing methods to increase ACE-Q response rates from both the patient and providers and evaluating the approaches used by sites with high ACE-Q response rates are important to maintain adequate screening measures.

### Potentials of Video-based Patient Education

Specifically for the ACE-Q form, there is a significant stigma surrounding the disclosure of difficult family circumstances, with associated feelings of parental guilt and shame and fears of Department of Children and Families involvement.^14^ Video-based educational interventions can be a standardized, well-organized way for providers to educate patients and their families on health topics and the need to collect certain information. It can also combat this stigma, as has been demonstrated with other health topics.^20^ Disclosure of ACEs is also a process, which is why regular re-screening and re-education over several well-child visits is needed to obtain the entire picture of toxic stress for ACE-positive children.^14^ Providers also need periodic reminders about the importance of consistent ACE-Q screening for this reason and for the sustainability of keeping up screening rates.

### Notes on Sustainability and Future Investigation

Sustainability is one concern in maintaining ACE-Q response rates and quality improvement. The preintervention for year 2 should ideally maintain the postintervention response rate of the year 1 intervention. However, in the chart review, the preintervention response rate in year 2 was less than the postintervention response rate in year 1, suggesting nonsustained changes in the period between PDSA cycles. In future investigations, a better understanding of the factors that help sustain and standardize changes in collecting important screener data will help to implement interventions that will continue to improve response rates throughout the year.

### Limitations

Our study has several limitations. First, few YouTube view counts for the video intervention indicated limited viewing. It would be important to investigate barriers to watching the video before beginning another study utilizing this media. A study across a larger number of clinics with higher view counts would be helpful in fully understanding the impact of a video education tool on the ACE-Q response rate.

An inherent limitation with a sensitive form, like the ACE-Q, is truthfulness and disclosures from caregivers. When dealing with sensitive information, some caretakers may not be open to disclosing abuse, neglect, or other household dysfunction that their child has experienced. We suspect that the caretakers who are most afraid of disclosing harmful childhood environments would be those of the children most at risk. We suspect that increased emphasis on ACE-Q form completion will lead to further conversations between the caregiver and the provider, which could cause visit time to increase. We did not assess for this measure in our study, but this would be an interesting variable to assess in future studies.

Additionally, because the interpretation and use of the ACE-Q score in the assessment and plan varied from provider to provider, it would be helpful to standardize the interpretation of the ACE-Q score for future research undertakings. One opportunity for standardization would be a template in the assessment and plan that allows providers to see suggested, peer-reviewed, and studied recommendations for patients depending on their ACE-Q score.

Finally, there was a period where no data were collected, making our project noncontinuous. This lapse in data collection was due to the project’s turnaround time, but it introduces limitations and potential biases. Because our data were collected during 2 separate cycles with a period of no data collection, this might introduce biases, particularly if certain times or events are underrepresented. This possibility might cause our data to not accurately reflect the full spectrum of variability in the system that we are studying. In a future, larger analysis of ACE-Q response rates, it would be important to consider continuous data collection for a longer period of time.

### Conclusions

Video interventions and personnel education positively affect ACE-Q response rates, though results vary from setting to setting. Consistent ACE-Q screening, documentation, and incorporation in assessments and plans are key to utilizing ACE-Q scores to prevent future negative health outcomes. Continuing to sustain these changes, reviewing and improving the accessibility of video education to patients, and working on new ways to increase ACE-Q screening, such as standardized note templates, is a focus for future investigation.^21^

## ACKNOWLEDGMENT

The authors would like to thank the Office of Research, Innovation, and Scholarly Endeavors at the University of South Florida Morsani College of Medicine for their assistance with data analysis.
